# Dosing of Ceftriaxone and Metronidazole for Children With Severe Acute Malnutrition

**DOI:** 10.1002/cpt.1078

**Published:** 2018-04-19

**Authors:** Joseph F. Standing, Martin O. Ongas, Caroline Ogwang, Nancy Kagwanja, Sheila Murunga, Shalton Mwaringa, Rehema Ali, Neema Mturi, Moline Timbwa, Christine Manyasi, Laura Mwalekwa, Victor L. Bandika, Bernhards Ogutu, Joseph Waichungo, Karin Kipper, James A. Berkley, for the FLACSAM‐PK Study Group

**Affiliations:** ^1^ Great Ormond Street Institute of Child Health University College London London UK; ^2^ Paediatric Infectious Diseases Research Group Institute for Infection and Immunity, St. George's, University of London London UK; ^3^ Center for Research in Therapeutic Sciences Strathmore University Nairobi Kenya; ^4^ KEMRI‐Centre for Clinical Research Nairobi Kenya; ^5^ KEMRI‐Wellcome Trust Research Programme Kilifi Kenya; ^6^ Mbagathi County Hospital Nairobi Kenya; ^7^ Coast General Hospital Mombasa Kenya; ^8^ Analytical Services International, St George's University of London London UK; ^9^ Institute of Chemistry University of Tartu Tartu Estonia; ^10^ The Childhood Acute Illness & Nutrition (CHAIN) Network Nairobi Kenya; ^11^ Centre for Tropical Medicine & Global Health, Nuffield Department of Medicine University of Oxford Oxford UK

## Abstract

Infants and young children with severe acute malnutrition (SAM) are treated with empiric broad‐spectrum antimicrobials. Parenteral ceftriaxone is currently a second‐line agent for invasive infection. Oral metronidazole principally targets small intestinal bacterial overgrowth. Children with SAM may have altered drug absorption, distribution, metabolism, and elimination. Population pharmacokinetics of ceftriaxone and metronidazole were studied, with the aim of recommending optimal dosing. Eighty‐one patients with SAM (aged 2–45 months) provided 234 postdose pharmacokinetic samples for total ceftriaxone, metronidazole, and hydroxymetronidazole. Ceftriaxone protein binding was also measured in 190 of these samples. A three‐compartment model adequately described free ceftriaxone, with a Michaelis–Menten model for concentration and albumin‐dependent protein binding. A one‐compartment model was used for both metronidazole and hydroxymetronidazole, with only 1% of hydroxymetronidazole predicted to be formed during first‐pass. Simulations showed 80 mg/kg once daily of ceftriaxone and 12.5 mg/kg twice daily of metronidazole were sufficient to reach therapeutic targets.

**Study Highlights**

**WHAT IS THE CURRENT KNOWLEDGE ON THE TOPIC?**

☑ There are no published pharmacokinetic data on ceftriaxone in infants and young children with severe acute malnutrition (SAM). A small study on oral metronidazole has been conducted, but no information on its major active metabolite is available. Changes in body composition and metabolism may affect the distribution and elimination of these agents. Inappropriate dosing may result in reduced efficacy, antimicrobial resistance, or increased adverse effects.

**WHAT QUESTION DID THIS STUDY ADDRESS?**

☑ What dose of ceftriaxone and metronidazole should be used in children with SAM who are sick and hospitalized?

**WHAT DOES THIS STUDY ADD TO OUR KNOWLEDGE?**

☑ A dose of 80 mg/kg of ceftriaxone (rather than 50 mg/kg) is predicted to maximize target attainment. Twice daily metronidazole at 12.5 mg/kg (rather than 7.5 mg/kg three times daily) may provide a good compromise between achieving circulating target concentrations, maintaining adequate small intestinal concentrations, and reducing caregiver time. Reduced serum albumin increased the unbound fraction of ceftriaxone. Edema significantly affected the disposition of both drugs, with weight‐based dosing leading to higher exposures, although this does not appear to warrant dose reduction.

**HOW MIGHT THIS CHANGE CLINICAL PHARMACOLOGY OR TRANSLATIONAL SCIENCE?**

☑ Our data inform optimal dosing in this vulnerable group of children. Careful prospective study design enables pharmacokinetic parameter estimation of two drugs using only three postdose samples.

In Sub‐Saharan Africa, infants and young children with complicated severe acute malnutrition (SAM) requiring hospital admission have a 12 to > 30% inpatient case fatality,^1^ with bacterial infection being a common cause. As a result, the World Health Organization (WHO) recommends that all children admitted to hospital with SAM receive broad‐spectrum antimicrobial treatment, usually with a combination of a beta‐lactam plus aminoglycoside antibiotics.^2^ Recent studies suggest increasing antimicrobial resistance but there is no evidence for the efficacy of alternative first‐line antimicrobial combinations in this context.^3^

Ceftriaxone, a broad‐spectrum third‐generation cephalosporin, is a potentially useful first‐line treatment for children with SAM. It is inexpensive and, due to its long half‐life, only requires once‐daily administration, which is important to overstretched healthcare settings where children with SAM are usually managed. When assessing its likely antimicrobial efficacy, the free concentration needs to be considered, since ceftriaxone protein binding is nonlinear, with higher total concentrations being associated with larger fraction unbound. Its nonlinear concentration effect is further compounded by increased free fraction with decreasing plasma proteins, most notably albumin. Models describing nonlinear ceftriaxone protein binding have been developed in special populations such those as in intensive care.^4^ SAM is associated with potentially reduced available plasma proteins, and often commonly presents with edema, both of which may affect ceftriaxone disposition.

In addition to sepsis, small intestinal bacterial overgrowth and intestinal parasitosis are implicated in malabsorption, diarrhea, and reduced nutritional recovery in children with SAM. Empiric oral metronidazole, a nitroimidazole antibacterial active against a wide range of bacteria including anaerobes, is advocated by some experts,^5^ and may aid long‐term growth recovery,^6^ but its efficacy for mortality and nutritional recovery are yet to be studied in an appropriately designed clinical trial. The pharmacokinetics (PK) of metronidazole in SAM may be altered with its half‐life prior to nutritional rehabilitation being reported in one study as up to 23 hours.^7^ Furthermore, its major hydroxy metabolite has around 65% activity compared with the parent drug.^8^ Hydroxymetronidazole pharmacokinetics have yet to be studied in children with SAM.

Here we report a study on ceftriaxone and metronidazole pharmacokinetics in order to define appropriate dosing for the sick, hospitalized infants and young children with SAM. This study forms the initial stage of the First Line Antimicrobials in Complicated Severe Acute Malnutrition (FLACSAM) trial (http://clinicaltrials.gov NCT03174236), which will compare ceftriaxone (±metronidazole) with standard‐of‐care antimicrobials (±metronidazole) in a 2 × 2 factorial design for outcomes including mortality and nutritional recovery. An important risk associated with unrestricted ceftriaxone use is selection for extended spectrum beta lactamase (ESBL) and resistance to other antibiotics classes. It will therefore be particularly important for the study of real‐world resistance development to be done in the context of optimally efficacious ceftriaxone dosing.

## RESULTS

### Patients and demographics

A total of 81 infants and young children with SAM were recruited in three centers in Kenya. A baseline sample was taken from all patients, and then 234 postdose samples were collected from which total ceftriaxone, metronidazole, and hydroxymetronidazole was measured. Unbound ceftriaxone was also measured in 190 of these samples. In 12 patients the baseline sample contained metronidazole and/or hydroxymetronidazole, and in one patient ceftriaxone was detected in the baseline sample. These patients were excluded from the pharmacokinetic analysis of the drug detected in their baseline sample, but retained for analysis of the other drug. Vomiting following one of the metronidazole doses occurred in seven patients, whereas two vomited following two of the doses. The total number of metronidazole doses during PK sampling was 572 giving a vomiting rate per dose of 1.6%. Demographics and clinical presentation are given in **Table** 1.

**Table 1:** Demographics of all patients, and those included in the ceftriaxone and metronidazole pharmacokinetic analyses

Variable	All patients (*n* = 81)	Included in ceftriaxone analysis (*n* = 80)	Included in metronidazole analysis (*n* = 69)
Age (mo)	14 (2–45)	15 (2–45)	17 (2–45)
Weight (kg)	5.88 (2.53–10.9)	5.88 (2.53–10.9)	5.88 (2.53–10.9)
Sex (m/f)	35/46	35/45	30/39
Height (cm)	69.4 (50.8–89)	69.7 (50.8–89)	69.4 (50.8–89)
Na + (mmol/L)	137 (109–165)	137 (109–165)	137 (109–165)
K + (mmol/L)	3.8 (0.7–7)	3.8 (0.7–7)	3.9 (0.7–7)
Hemoglobin (g/dL)	9 (4.4–12.8)	9 (4.4–12.8)	9 (4.4–12.8)
WBC (x10^9^/L)	14.1 (4.8–58.1)	14.35 (4.8–58.1)	13.5 (4.8–58.1)
Neutrophils (x10^9^/L)	5.27 (1.2–66.4)	5.4 (1.2–66.4)	5.27 (1.2–66.4)
Lymphocytes (x10^9^/L)	5.39 (1.5–17.82)	5.36 (1.5–17.82)	5.3 (1.5–17.82)
Platelets (x10^9^/L)	397 (19–775)	398 (19–775)	375 (19–775)
Albumin (g/L)	33.6 (7–57)	33.75 (7–57)	30.7 (7–57)
Total protein (g/L)	59 (11–92)	59 (11–92)	58 (11–90)
Creatinine (micromol/L)	21 (4–178)	21 (4–178)	20 (4–178)
AST (IU/L)	53 (6–758)	53 (6–758)	52 (6–270)
Edema grade (0/1/2/3)	45/13/14/9	45/12/14/9	37/11/13/8
Median weight by edema grade (kg)	5.18/5.88/8/8.13	5.18/5.88/8/8.13	5.18/5.88/8.14/7.96

Median and range are presented for continuous variables.

### Pharmacokinetic structural models

A three‐compartment model provided a slightly better fit than a two‐compartment model (⊿ OFV −7.4) to total ceftriaxone, with unbound measurements predicted assuming instantaneous binding with a Michaelis–Menten model (see **Methods**). For the simultaneous modeling of metronidazole and hydroxymetronidazole, a one‐compartment disposition model was adequate for both, and adding a zero order lag to the first order absorption gave a significant improvement in fit (*P* < 0.05). Allometric scaling^9^ was added *a priori* along with a fixed sigmoidal maturation function on clearance based on glomerular filtration maturation for ceftriaxone^10^ and CYP2A6 maturation^11^ for metronidazole. Neither maturation model improved fit (⊿ OFV < 3.84), but nor was the fit worsened, and so they were retained in the model as biological priors for extrapolation. In the case of metronidazole, initial model building excluded patients who vomited within 2 hours of a dose, but including these patients did not noticeably change the goodness‐of‐fit, and the largest change in parameter estimate was a 15% decrease in clearance. Since one would expect lost dosing due to vomiting if anything to increase apparent clearance, it was decided to retain these samples. Hydroxymetronidazole was described using a semimechanistic model allowing formation from both first‐pass and circulating drug, the model with only formation from circulating drug giving a significantly worse fit (*P* < 0.001). A low extraction ratio of around 1% indicated most hydroxymetronidazole is formed from systemic metronidazole. Both models terminated with successful covariance steps, and model parameters are reported in **Table** 2. NONMEM code and model diagnostics for both models are provided in the **Supplementary Materials**.

**Table 2:** Parameter estimates scaled to a 70 kg individual from the ceftriaxone and metronidazole models. Pharmacokinetic parameters for ceftriaxone relate to free concentrations, except V1 which is the total ceftriaxone central volume. CLIMET is the metronidazole intrinsic clearance to hydroxymetronidazole, CLMET is the apparent clearance of metronidazole by other routes, and CLOH the clearance of hydroxymetronidazole.

Parameter	Estimate (bootstrap 95% CI)	IIV (bootstrap 95% CI)	Shrinkage (%)
**Ceftriaxone**			
CL (L/h)	4.62 (4.06,5.24)	42 (33,49)	3.3
V1 (L)	14.46 (9.47,17.76)	43 (28,71)	17.9
	0.93 (0.16,5.61)	—	—
Q2 (L/h)	6.05 (2.01,18.67)	—	—
V2 (L)	29.98 (20.32,68.15)	—	—
Q3 (L/h)	19.26 (12.39,25.98)	—	—
V3 (L)	69.77 (54.65,87.64)	42 (26,55)	17.1
B_M_ (mg/h)	22.89 (17.43,30.57)	—	—
K_D_ (mg/L)	0.56 (0.21,0.92)	—	—
θ_ALB_	–0.26 (–0.41,–0.12)	—	—
θ_SECR_	–0.26 (–0.36,–0.14)	—	—
θ_ODEM_CEF_	27 (19,33)	—	21.4
σ_PROP_FREE_ (%)	19 (15,22)	—	21.0
σ_PROP_TOT_ (%)	4.62 (4.06,5.24)	42 (33,49)	3.3
σ_ADD_TOT_ (mg/L)	14.46 (9.47,17.76)	43 (28,71)	17.9
**Metronidazole**			
Ka (/h)	0.39 (0.29,0.6)	79 (63,97)	13.9
CLI_MET_ (L/h)	0.74 (0.51,1.08)	61 (47,76)	5.2
CL_MET_ (L/h)	0.51 (0.23,0.76)	—	—
V_MET_ (L)	56.35 (47.73,65.48)	38 (27,45)	18.3
CL_OH_ (L/h)	1.45 (1.08,1.95)	—	—
V_OH_ (L)	16.89 (11.67,24.77)	—	—
Alag (h)	0.17 (0.11,0.26)	—	—
θ_ODEM_MET_	–0.17 (–0.31,–0.01)	—	—
σ_PROP_MET_ (%)	32 (27,37)	—	17.7
σ_PROP_OHMET_ (%)	29 (24,33)	—	17.7
σ_PROP_OHADD_ (mmol/L)	0.01 (0.002,0.04)	—	17.7

Covariates entered the model as follows: B_M_ = TV_BM_(Alb/33.75)^θALB^ where Alb the individual albumin and 33.75 the median; CL = TVCL(SCR/MSCR)^θSECR^ where SCR is the individual serum creatinine, and MSCR the expected serum creatinine for age; WTC = WT(1 + θ_ODEM_CEF_ or θ_ODEM_MET_) where WTC is the corrected weight for patients with edema used in the allometric scaling model. NONMEM code is provided in the **Supplementary Material**.

### Edema significantly affects disposition

For both ceftriaxone and metronidazole, adding edema as a covariate on body weight in the allometric model for volume improved fit. In the case of ceftriaxone, the largest drop in objective function (OFV) was associated with decreased apparent weight on volume parameters in the allometric model with any grade of edema of 26%. For metronidazole the effect of edema was less pronounced, with the largest OFV drop occurring with an edema score of 2 or more leading to a decrease in apparent weight of 17%.

### Ceftriaxone clearance associated with renal function and protein binding predicted from serum albumin

**Figure 1:**
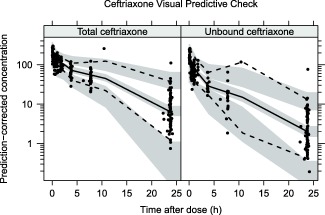
Prediction corrected visual predictive check (VPC) for total and unbound ceftriaxone (top row) showing model simulated 95% confidence intervals for the simulated 2.5th, 50th, and 97.5th percentiles.

**Figure 2:**
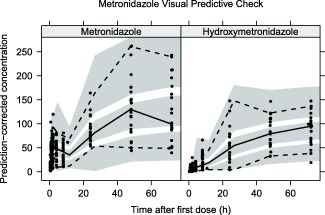
Prediction corrected visual predictive check (VPC) for metronidazole and hydroxymetronidazole showing model simulated 95% confidence intervals for the simulated 2.5th, 50th, and 97.5th percentiles.

Free ceftriaxone clearance was significantly (*P* < 0.001) associated with age‐corrected serum creatinine.^12^ Free ceftriaxone was modeled directly, with total ceftriaxone predicted by assuming instantaneous binding and a concentration‐dependent Michaelis–Menten model. Albumin was found to significantly (*P* < 0.001) improve the fit when added to the *B*_*max*_ parameter in the Michaelis–Menten model, with lower serum albumin being associated with higher fraction unbound. **Figures** 1 and 2 show visual predictive checks for the ceftriaxone and metronidazole final models, respectively. Basic goodness‐of‐fit plots, a plot of fraction unbound vs. total concentration with different albumin concentrations, and a schematic of the final model is provided in the **Supplementary Materials**.

### Probability of target attainment to inform dosing

**Figure 3:**
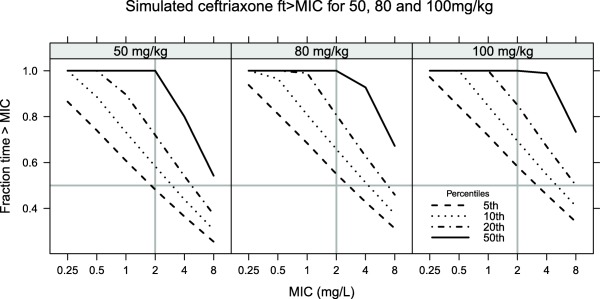
*ft* > *MIC* for 50, 80, and 100 mg/kg once daily. The 5th, 10th, 20th, and 50th percentiles show the cutoffs for *ft* > *MIC* for 95, 90, 80, and 50% of patients, respectively. Lines at a MIC cutoff of 2 mg/L and *ft* > *MIC* of 0.5 show the proposed MIC target.

**Figure 4:**
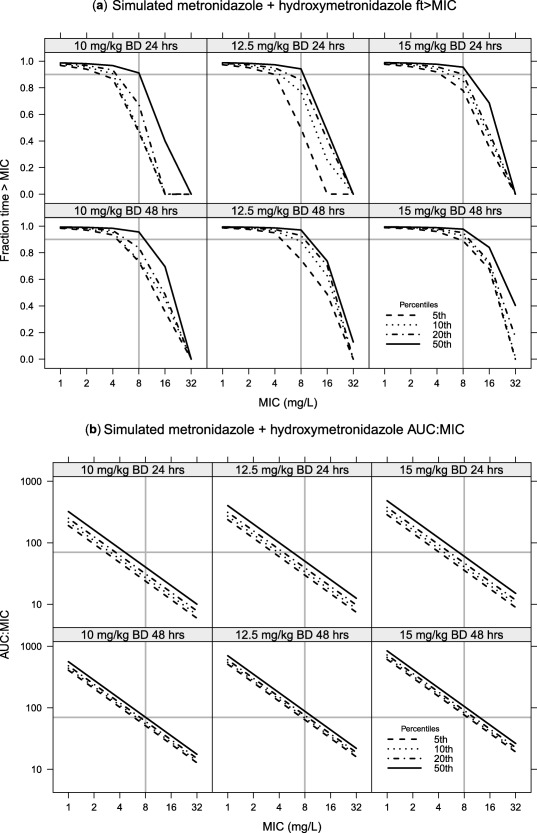
Simulations for free metronidazole plus hydroxymetronidazole under 10, 12.5, and 15 mg/kg twice daily dosing, with hydroxymetronidazole concentrations being multiplied by 0.65 to reflect the assumed lower activity. A break‐point of 8 mg/L is highlighted. (**a**) Yhe *ft* > *MIC* with a value of 0.9 highlighted; whereas (**b**) shows the AUC:MIC ratio with a cutoff of 70 mg.h/L highlighted. A comparison of 24‐ and 48‐hour target attainment is given in the upper and lower panels, respectively.

**Figure 5:**
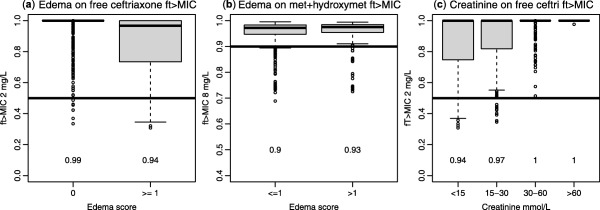
Boxplots to illustrate simulated covariate effects on translation to *ft* > *MIC*. Probability of target attainment reported beneath each box with a target of 50% of the dosing interval with concentrations above 2 mg/L at 24 hours for free ceftriaxone, and 90% of the dosing interval with concentrations of free metronidazole and 0.65 x hydroxymetronidazole above 8 mg/L at 48 hours. (**a**) Effect of edema score of 1 or more on ceftriaxone target attainment. (**b**) Effect of edema score of 2 or more on metronidazole target attainment. (**c**) Effect of serum creatinine on ceftriaxone target attainment.

Fraction time above MIC (*ft* > *MIC*) for ceftriaxone free concentration over the first 24 hours was simulated for 50, 80, and 100 mg/kg once daily dosing (**Figure** 3). For metronidazole the sum of the free concentration and 0.65 multiplied by the free hydroxymetronidazole concentration was used to simulate twice‐daily dosing regimens of 10, 12.5, and 15 mg/kg with *ft* > *MIC* and AUC:MIC ratio calculated for the 24 hour periods 0–24 hours and 24–48 hours (**Figure** 4). The effects of included covariates on pharmacodynamic target attainment are presented in **Figure** 5.

## DISCUSSION

This is, to our knowledge, the first detailed pharmacokinetic study of ceftriaxone in infants and young children with SAM,^3^ and we have also augmented the limited available data on oral metronidazole pharmacokinetics in this population.^7^ Edema, a common feature of SAM, significantly affected the disposition of ceftriaxone, and to a lesser extent metronidazole, with patients having a lower volume of distribution than would be expected from their total body weight. This did not translate into marked changes in circulating concentrations or produce a clear indication of the need for dose reduction (**Figure** 5), indicating clearance is more important for *ft* > *MIC* than volume of distribution in this case. While no other covariates were significant for metronidazole, in common with previous studies we found decreased ceftriaxone clearance with worsening renal function^13^ and also, as expected, the fraction unbound significantly related to serum albumin.^4^ Again, as expected,^14^ changes in albumin had no effect on free drug concentration or target attainment. As in previous studies,^15^, ^16^, ^17^ rather than use a model‐based estimate of creatinine clearance we used serum creatinine scaled by the age appropriate typical value^12^ to identify patients deviating from an expected normal value. This is a similar principle to using a creatinine clearance value scaled to 1.73 m^2^ but that approach only delineates size‐related changes in expected creatinine clearance, not age. In terms of the impact of covariates on target attainment, simulations showed that albumin was not an important predictor of free ceftriaxone, whereas patients with low serum creatinine had reduced ceftriaxone target attainment (**Figure** 5). The degree of this reduction, however, was not sufficient to necessitate dose increases in these patients.

In this study precise pharmacokinetic parameter estimates were obtained with low shrinkage (see **Table** 2) 18 using only three postdose samples. This is due to a careful sampling schedule, which although not defined with optimal design methods, followed the general principle of sampling close to the likely *C*_*max*_ to give information on distribution, late samples aiming to cover at least three expected half‐lives to inform on clearance, and samples in the expected absorption phase for oral metronidazole. By randomizing patients to different combinations of early, middle, and late sampling points, in a similar manner to a previous antimicrobial study in this population,^19^ study invasiveness was minimized while maximizing information content. This approach exemplifies the benefits of careful study design, avoiding the problems that can be caused with opportunistic and scavenged sampling.^20^

Unlike some ceftriaxone pharmacokinetic studies,^21^ we measured both total and free ceftriaxone from patient samples, and under the assumption that only free ceftriaxone was available for distribution, elimination, and antimicrobial activity, it was modeled directly rather than being inferred from total concentrations.^4^ Due to the expense of measuring free ceftriaxone, it was not performed in every sample and where possible we aimed to obtain free concentrations from an early and a late sample in order to maximize data on the concentration range from that individual. Previous studies have not directly modeled free ceftriaxone; rather, the tendency has been to model total ceftriaxone and infer free concentrations from this. If only free ceftriaxone is available for distribution and elimination, modeling total ceftriaxone would appear to be problematic, since its nonlinear protein binding means volume of distribution will change with total concentration. We used a method similar to that of a recent study on cefazolin,^24^ whereby total concentration in the central compartment was used to predict free concentration and free fraction, and it was only the free fraction that was available for elimination and distribution. Full details of the model, including NONMEM code, are provided in the **Supplementary Material**.

Comparing our model parameters with other studies is complicated by many reporting parameters for total ceftriaxone and a lack of standardization of age and weight (or size) scaling.^9^ Our free ceftriaxone clearance estimate standardized to a 70 kg mature individual with normal creatinine for age and gender was 4.62 L/h. This estimate is slightly lower than patients without renal impairment in a recent report of adult intensive care patients (6.2 L/h), higher than the neonatal‐reported value of 1.61 L/h/1.73 m^2^,^25^ but lower than the 14.86 L/h reported in healthy adult volunteers.^26^ Regarding protein binding, our dissociation constant (*K*_*d*_) of 22.9 mg/L is similar to the value (28.7 mg/L) that was recently reported by Schleibinger *et al*.^4^ No effect of adding maturation functions to clearance parameters in either the ceftriaxone or metronidazole models was found, most likely because most of our patients would be predicted close to the mature value by their age and we only had data in the upper part of the maturation curve. For example, our youngest patient had a postmenstrual age of 46 weeks, which is approximately the half‐mature value in the renal clearance model.^10^ The rationale for retaining maturation functions is to provide biological prior information in case the models were to be used for extrapolation to younger patients.

When using the model for simulation of pharmacodynamic target attainment, we chose not to follow the traditional method of defining a single target and simulating the probability of target attainment (PTA) against ascending MIC values. Our reasons for this are twofold: first, such plots tend to give measures of spread of target attainment across the population; second, the preferred target may not be fixed, with recent work showing that different populations may require different *ft* > *MIC* values.^27^ We therefore chose to plot the actual target (*ft* > *MIC* or *AUC:MIC*) with ascending MIC values, along with important percentiles from the simulations. This therefore shows not just the simulated typical patient, but, for example, the 5th percentile shows the minimum target attainment expected in 95% of patients. We would suggest that presenting antimicrobial simulation data in this way (**Figures** 3 and 4) provides more information than the traditional PTA curve.

By parameterizing the model in terms of free ceftriaxone, simulations of free concentrations were therefore straightforward, and the *ft* > *MIC*, which is the index usually found to correlate with antimicrobial activity,^28^ was simulated for a range of MIC values (**Figure** 3). A common target for beta lactam antimicrobials is to achieve a *ft* > *MIC* of at least 50% of the dosing interval, and typically *Enterobacteriaciae* with MICs below 1 mg/L, were considered susceptible, although 2 mg/L is now a more common breakpoint, with MIC >8 mg/L indicating the need to test for the presence of ESBL.^29^ Our simulations show that for 50 mg/kg dosing there is a greater than 95% probability of having 50% of the dose interval with concentrations exceeding 2 mg/L and increasing the dose to 80 mg/kg improves this to 95% of patients of patients having greater than 60% of the dose interval exceeding 2 mg/L, and greater than 80% of patients achieving 50% above 8 mg/L (the ESBL breakpoint).

Including metronidazole formation from first‐pass and circulating concentrations gave a 70.8 point lower objective function value than when only circulating concentrations were allowed to form metabolite, and hence it was retained despite the small fraction (around 1%) formed in this way. In the standard parent metabolite model, allowing formation from circulation and first‐pass is not uniquely identifiable since the formation rate cannot be separated from the fraction metabolized.^30^ Using the well‐stirred hepatic metabolism model, the formation rate is bounded by liver plasma flow, and hence the intrinsic clearance parameter is the only parameter estimated. This is a similar approach to that taken in a study of oseltamivir carboxylate formation from oseltamivir,^31^ and gave a good description of our data (see **Figure** 2). Without intravenous data the model parameters estimated, however, are still apparent, since the total bioavailability is not identifiable. An investigation of parameter identifiability and parameter interpretation is provided in the **Supplementary Materials**. Including hydroxymetronidazole was important, since it is known to have antimicrobial activity in its own right.^32^ Our decision to sample patients up to 72 hours post first‐dose based on previous data in this population^7^ was clearly justified, as accumulation can be seen for both metronidazole and hydroxymetronidazole (**Figure** 2).

The pharmacodynamic target of metronidazole in this setting is somewhat unclear. While time above an MIC cutoff of 8 mg/L^33^ or AUC(0‐24)/MIC of 70^8^ have been proposed, and could potentially be used to justify once‐daily dosing along with the use of a loading dose, when treating small intestinal overgrowth it could be argued that local upper gastrointestinal tract concentrations maybe more important for efficacy. Taking our estimated absorption rate constant and assuming a gut lumen volume of 100 mL (dose taken with food/drink) a 12.5 mg/kg dose administered to an 8‐kg child would leave a local concentration of 7.3 mg/L prior to the next dose. Twice‐daily dosing would also give acceptable AUC and *ft* > *MIC* target attainment (**Figure** 4). Although twice‐daily dosing requires higher volumes of suspension to be administered, only 1.6% of doses in this study were followed by the patient vomiting. It should, however, be noted that we did not find vomiting within 2 hours of a dose to affect metronidazole PK estimates when they were included in the analysis. A dose of 12.5 mg/kg twice daily gives a total daily dose of 25 mg/kg, which is slightly higher than the current recommended dose (7.5 mg/kg three times, giving 22.5 mg/kg/day).

In conclusion, for children who are severely ill with complicated SAM, this study supports doses of i.v. ceftriaxone 80 mg/kg once daily, which is in line with recent WHO recommendations. Our suggested dosing of oral metronidazole 12.5 mg/kg twice daily provides a simplified regime that should maintain efficacious upper gastrointestinal tract concentrations.

## METHODS

### Patient recruitment

A three‐center open‐label phase II PK study was undertaken in children aged 2–59 months with severe acute malnutrition (SAM). For children aged 6–59 months, SAM was defined as kwashiorkor (edematous malnutrition) or a middle upper arm circumference (MUAC) less than 11.5 cm, or weight‐for‐height Z‐score of less than –3. For children aged 1–5 months SAM was defined as kwashiorkor or MUAC less than 11 cm or weight‐for‐height Z score less than –3. Infants with weight less than 2.5 kg were excluded. The study received favorable ethical review from the Kenya Medical Research Institute Review Committee, Nairobi, the Oxford Tropical Research Ethics Committee, Oxford, UK, and the Kenyan Expert Committee on Clinical Trials of the Pharmacy & Poisons Board, and is registered with http://clinicaltrials.gov (NCT02746276). Parents of eligible patients were approached for informed consent.

### Drug administration, sampling, and assay

Enrolled patients received ceftriaxone 50 mg/kg (or 100 mg/kg if meningitis suspected) 24 hourly by intravenous bolus (250 mg or 500 mg vials, Roche, Nutley, NJ), and metronidazole 7.5 mg/kg 8 hourly by oral suspension (200 mg/5mL, Sanofi, Bridgewater, NJ). PK samples were taken up to 72 hours following the first dose. A baseline PK sample was taken and then the first metronidazole dose followed by the first ceftriaxone dose. To minimize study invasiveness, only three further PK samples were taken. Patients were randomized to one of three possible early, middle, and late samples as follows: Early: 5 minutes, 30 minutes, or 1 hour post first dose; Middle: 2 hours, 4 hours, or 8 hours post first dose; Late: 24 hours, 48 hours, or 72 hours post first dose with exact dosing and sampling time recorded to the nearest minute.

Samples were analyzed for total ceftriaxone, metronidazole, and hydroxymetronidazole. Free ceftriaxone was also measured in a subset of samples, with preference given to the early and late timepoints. Full details on the assay were recently published by Ongas *et al*.^35^ Briefly, a reversed‐phase high‐performance liquid chromatography method coupled with electrospray ionization mass spectrometry (HPLC–ESI‐MS/MS) method was developed requiring 50 μL of plasma for simultaneous ceftriaxone, metronidazole, and hydroxymetronidazole quantification (a further 25 μL was required for ultrafiltration and free ceftriaxone quantification). The intra‐ and interrun precision was less than 9% and the lower limits of quantification were 0.4, 0.05, and 0.02 μg/mL for ceftriaxone, metronidazole, and hydroxymetronidazole, respectively.

### Pharmacokinetic model building

Unbound ceftriaxone was first analyzed alone to derive a base structural disposition model, with one‐, two‐, and three‐compartment disposition models tested. It was assumed that bound drug is unavailable for elimination, distribution, or pharmacological effect, so when total concentrations were added, free concentration (*C*_*U*_) was predicted from total concentration (*C*_*T*_) in the central compartment, with bound concentration (*C*_*B*_) assumed to follow Michaelis–Menten kinetics with instantaneous binding:

$$C_{B}=\frac{B_{max}C_{U}}{K_{d}+C_{U}}$$

where *B*_*max*_ and *K*_*d*_ are the estimated Michaelis–Menten parameters, and $C_{T}=C_{U}+C_{B}$. The unbound concentration was therefore derived from the Michaelis–Menten equation above:

$$C_{U}=\frac{1}{2}\left(\left(C_{T}-B_{max}-K_{d}\right)+\sqrt{{\left(C_{T}-B_{max}-K_{d}\right)}^{2}+4K_{d}C_{T}}\right)$$

Metronidazole and hydroxymetronidazole were simultaneously fitted using the well‐stirred liver model, using a similar model to previous study on oseltamivir carboxylate formation from oseltamivir.^31^ A common absorption rate constant and fixed hepatic plasma flow were used to allow for both first‐pass and hydroxymetronidazole formation from circulating drug to be identified. Structural and practical model identifiability is discussed in the **Supplementary Material**. Hepatic plasma flow was derived from an allometrically scaled adult hepatic blood flow (73 L/h) scaled by measured hematocrit according to the expression reported by Price *et al*.^36^:

$$Q_{HP}=76{\left(\frac{w}{70}\right)}^{0.75}\left(1-h0.91\right)$$

where *Q*_*HP*_ is hepatic plasma flow, w is body weight in kg, and h is the hematocrit. The fraction transformed to hydroxymetronidazole during first‐pass was assumed to be 1 minus the extraction ratio (*E*_*R*_) and hepatic clearance of circulating metronidazole to hydroxymetronidazole was given by the following:

$$CL_{M}=Q_{HP}E_{R}=Q_{HP}\frac{CL_{I}}{CL_{I}+Q_{HP}}$$

where the intrinsic clearance, *CL*_*I*_, was an estimated parameter. Clearance of metronidazole by other routes was assumed to be linear, as was clearance of hydroxymetronidazole.

Biological prior information in the form of allometric scaling with exponents of 0.75, 1, and –0.25 for all clearance volume and absorption rate parameters, respectively, was added *a priori*. Furthermore, a maturation function for renal clearance based on a previous study^10^ was used to scale free ceftriaxone clearance and hydroxymetronidazole clearance. Metronidazole hydroxylation at the 2 position is catalyzed by CYP2A6,^37^ and hence the CYP2A6 maturation function reported by Upreti *et al*.^11^ was used to scale *CL*_*I*_.

Further covariates tested in the following order were: albumin and total plasma protein on *B*_*max*_, age corrected serum creatinine on free ceftriaxone clearance and hydroxymetronidazole clearance, and edema to scale weight in the allometric models. The likelihood ratio test was used to determine covariate inclusion, with an improvement in –2 log likelihood of > 3.84 considered significant. Model diagnostics included plots of observations vs. population predictions, conditional weighted residuals (CWRES) vs. time and prediction, and individual profile plots. Simulation properties were tested with a visual predictive check (VPC). Parameter stability was investigated using a nonparametric bootstrap. Modeling was undertaken using NONMEM v. 7.3^38^ using the first‐order conditional estimation algorithm with interaction (FOCEI). Perl‐speaks NONMEM (PsN) was used for VPC and bootstrap preparation,^39^ and data manipulations and plotting were performed using R v. 3.2.

### Pharmacodynamic simulations

A simulated population of 1,000 subjects with demographics sampled from the study population was generated and the ceftriaxone model was used to simulate the *ft* > *MIC* for MIC values in the range 0.25–8 mg/L. The 5th, 10th, 20th, and 50th percentiles were presented to show the minimum *ft* > *MIC* for 95, 90, 80, and 50% of patients, respectively. A target of 0.5 for *ft* > *MIC* with a MIC cutoff of 2 mg/L (the current EUCAST resistance breakpoint) was considered. For metronidazole, a pharmacodynamic target of area under the curve to MIC ratio AUC(0‐24)/MIC of 70^8^ and the *ft* > *MIC* of 8 mg/L were investigated.^33^ Simulations for once, twice, and three‐times daily dosing were performed, along with consideration of a loading dose. In these simulations 10% protein binding was assumed for parent and metabolite, and hydroxymetronidazole concentrations multiplied by 0.65 were added to metronidazole under the assumption of hydroxymetronidazole having 65% of its parent's antimicrobial activity.^32^ The results of all simulations for ceftriaxone and metronidazole were presented in a report to the FLACSAM trial steering committee to agree on dosing for the main trial.

## FUNDING

The FLACSAM trial, including this PK study, is funded by the United Kingdom Medical Research Council/Department for International Development/Wellcome Trust Joint Global Health Trials Scheme [MR/M007367/1]. J.F.S. was supported by a United Kingdom Medical Research Council Fellowship (grant MR/M008665/1) and at institution level by the National Institute for Health Research Biomedical Research Centre at Great Ormond Street Hospital for Children NHS Foundation Trust and University College London. J.A.B is supported by the United Kingdom Medical Research Council/Department for International Development/Wellcome Trust Joint Global Health Trials Scheme [MR/M007367/1] and the Bill & Melinda Gates Foundation for the Childhood Acute Illness & Nutrition Network (CHAIN) [OPP1131320].

## CONFLICT OF INTEREST

The authors declare no competing interests for this work.

## AUTHOR CONTRIBUTIONS

J.F.S. and J.A.B. wrote the article; J.A.B., M.O.O., B.O., J.F.S., and K.K. designed the research; C.A.O., R.A., N.M., N.K., M.T., C.M., L.M., V.L.B., S.M., M.O.O., and K.K. performed the research; J.F.S., S.M., M.O.O., and J.A.B. analyzed the data.

## Supporting information

Supplementary Material
