# Supplementary material for: Dosing of Ceftriaxone and Metronidazole for Children With Severe Acute Malnutrition
Source: Clin Pharmacol Ther. 2018 Apr 19;104(6):1165–74. doi: 10.1002/cpt.1078 (PMC6282491; doi:10.1002/cpt.1078)
Supplement: Supplementary file 1 — Supplementary Material [file CPT-104-1165-s001.pdf]

SUPPLEMENTARY MATERIAL: Dosing of ceftriaxone and metronidazole in infants with severe acute malnutrition

Contents

1 Supplementary figures **2**
1.1 Ceftriaxone basic goodness-of-fit plots . . . 2
1.2 Metronidazole basic goodness-of-fit plots . . . 6
1.3 Covariate correlations . . . 10
1.4 Relationship between albumin and ceftriaxone protein binding . . . 11
1.5 Ceftriaxone model details . . . 12

2 NONMEM model files **13**
2.1 Ceftriaxone NONMEM model code . . . 13
2.2 Metronidazole NONMEM model code . . . 15

3 Metronidazole model identifiability and parameter interpretation **16**
3.1 Structural model identifiability . . . 17
3.2 Practical identifiability . . . 18

1 Supplementary figures

1.1 Ceftriaxone basic goodness-of-fit plots

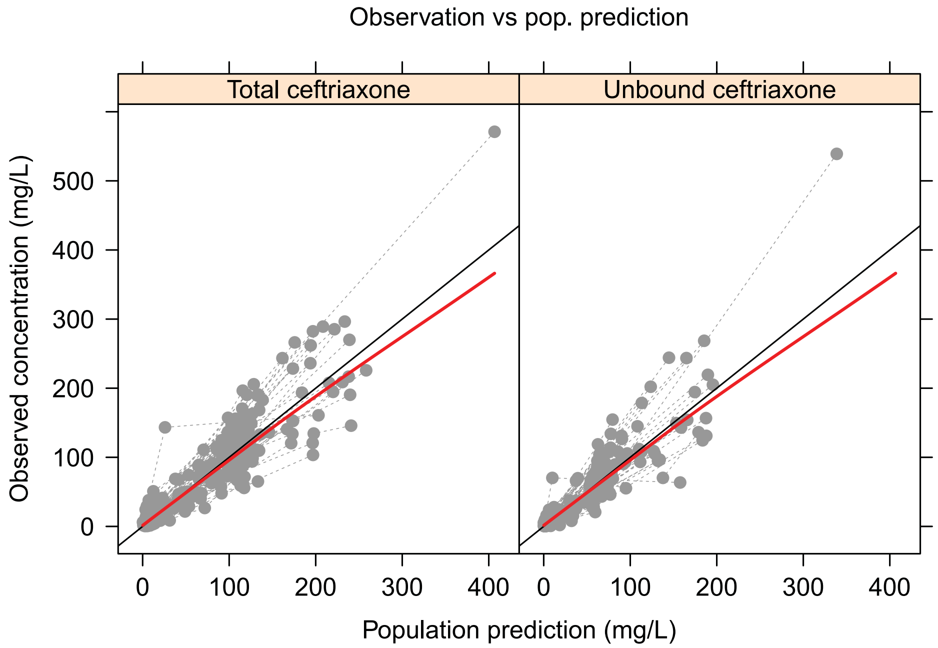

Figure S1: Population predictions plotted against observed concentrations for total and unbound ceftriaxone

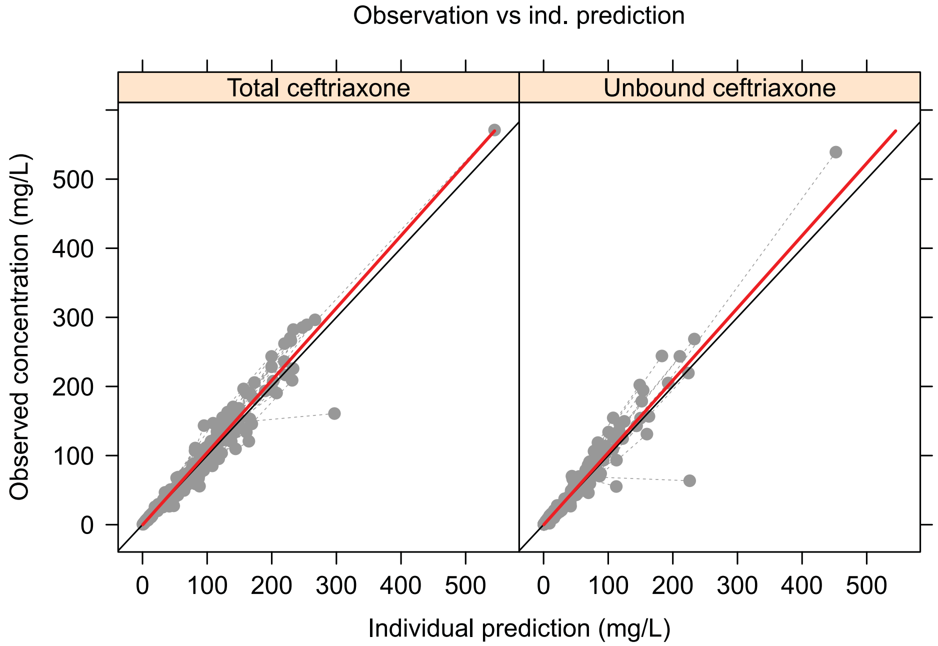

Figure S2: Individual predictions plotted against observed concentrations for total and unbound ceftriaxone

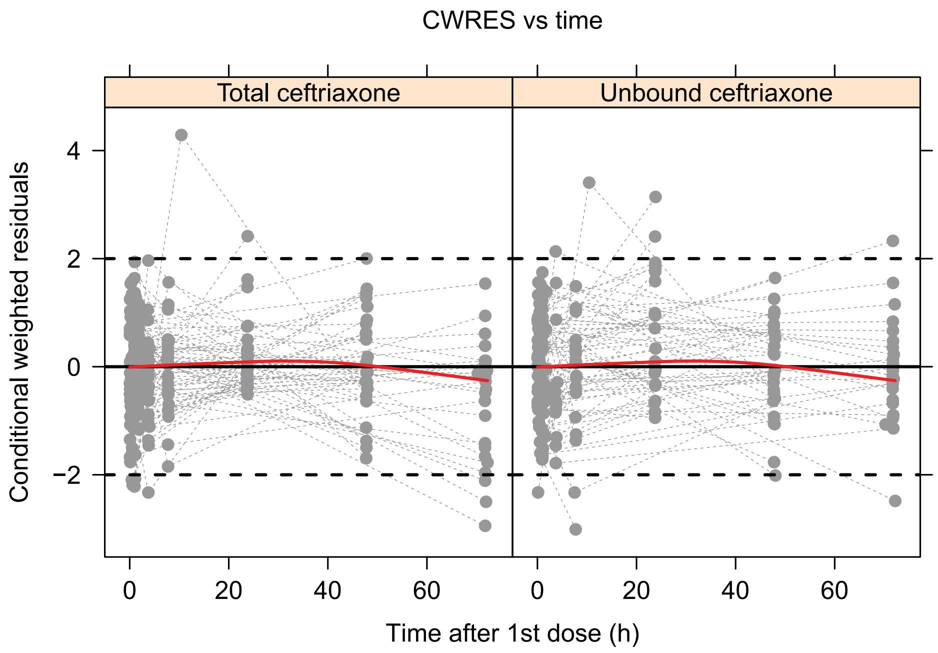

Figure S3: Conditional weighted resuduals versus time after first dose for total and unbound ceftriaxone

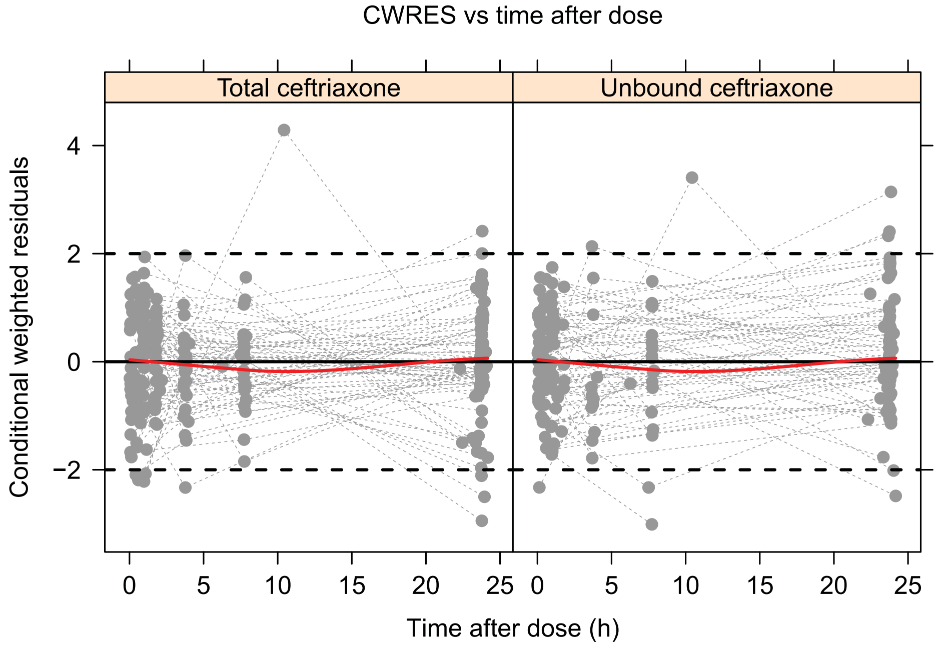

Figure S4: Conditional weighted resuduals versus time after dose for total and unbound ceftriaxone

1.2 Metronidazole basic goodness-of-fit plots

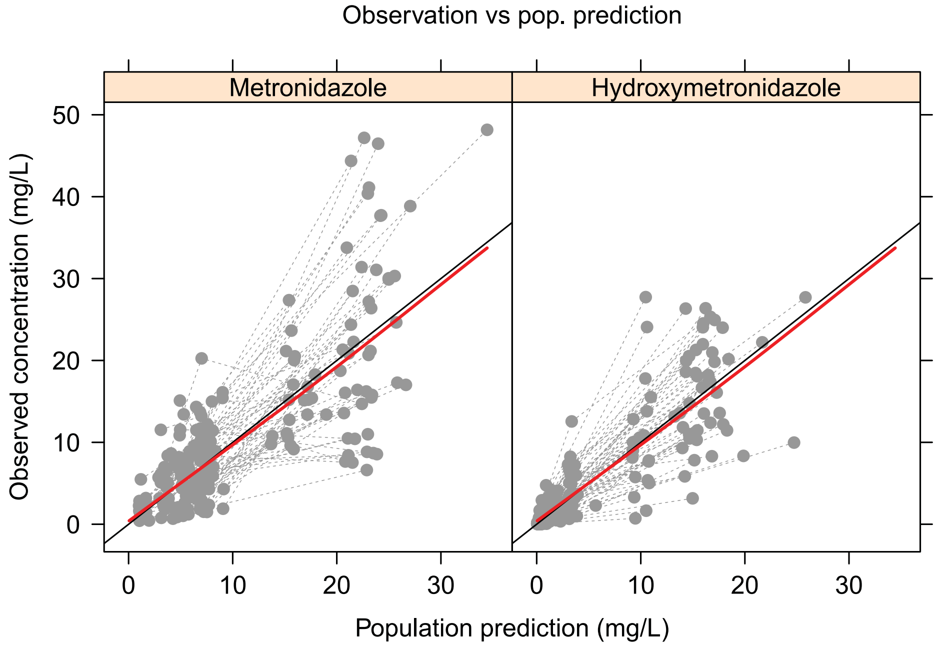

Figure S5: Population predictions plotted against observed concentrations for metronidazole and hydroxymetronidazole

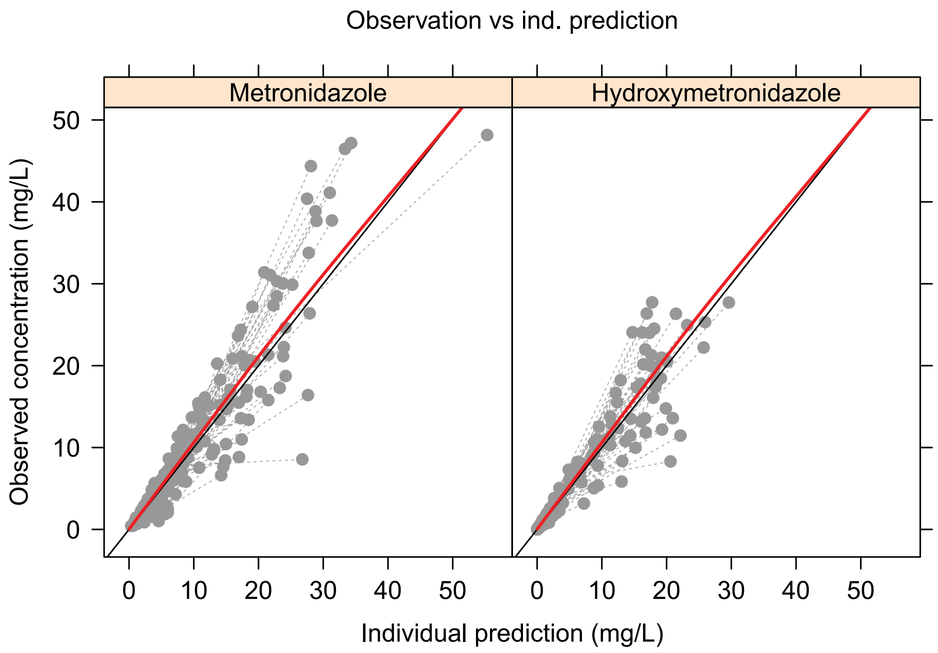

Figure S6: Individual predictions plotted against observed concentrations for metronidazole and hydroxymetronidazole

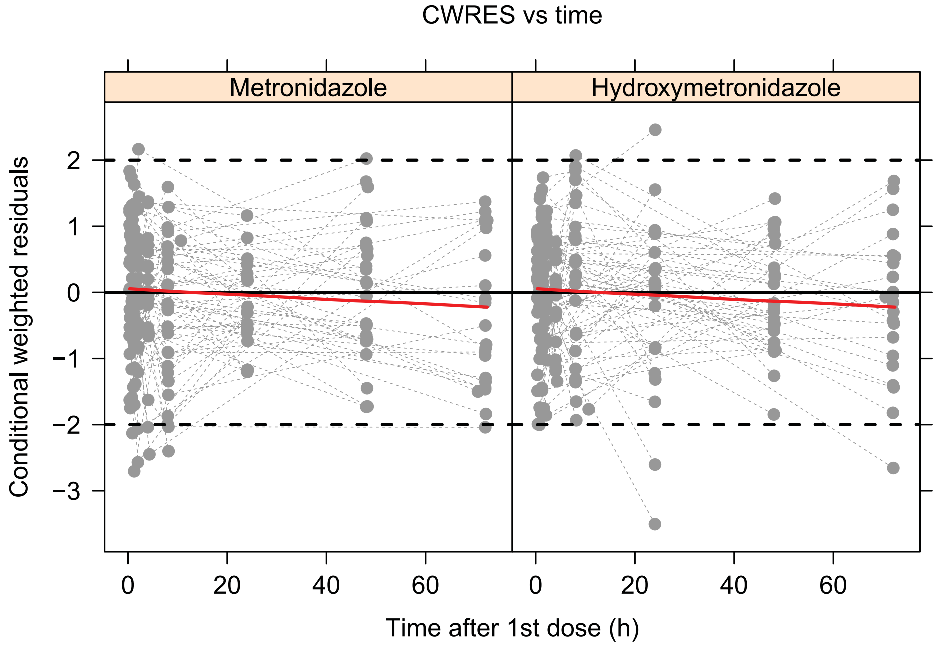

Figure S7: Conditional weighted resuduals versus time after first dose for metronidazole and hydroxymetronidazole

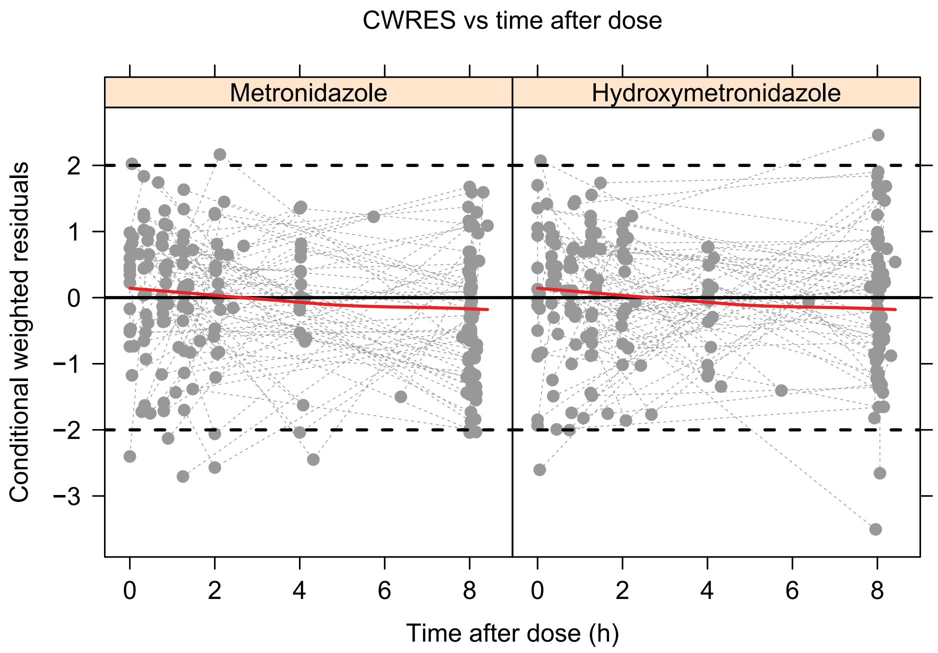

Figure S8: Conditional weighted resuduals versus time after dose for metronidazole and hydroxymetronidazole

1.3 Covariate correlations

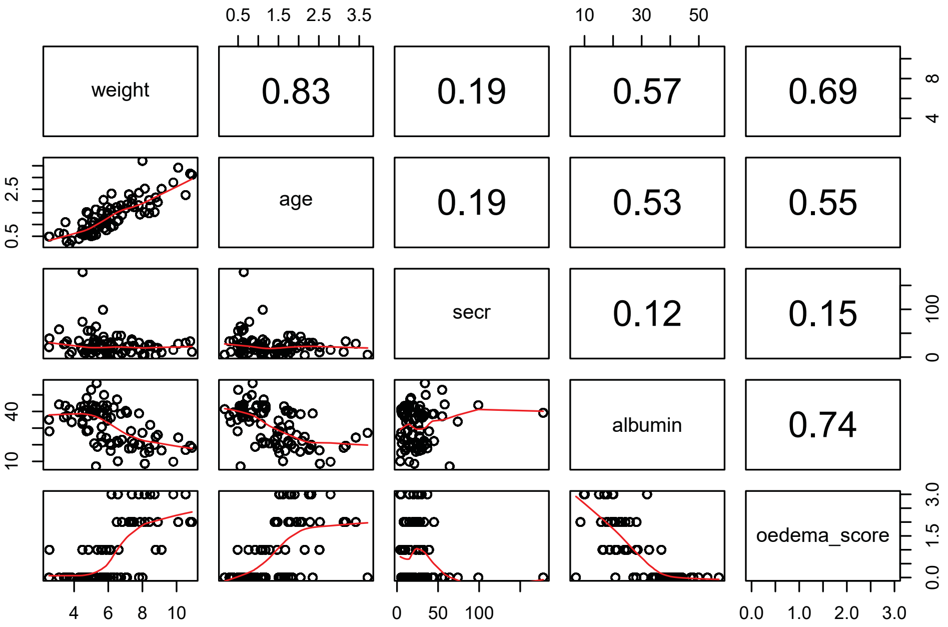

Figure S9: Correlations in covariates, plots in lower triangle, correlation coefficients in upper triangle

1.4 Relationship between albumin and ceftriaxone protein binding

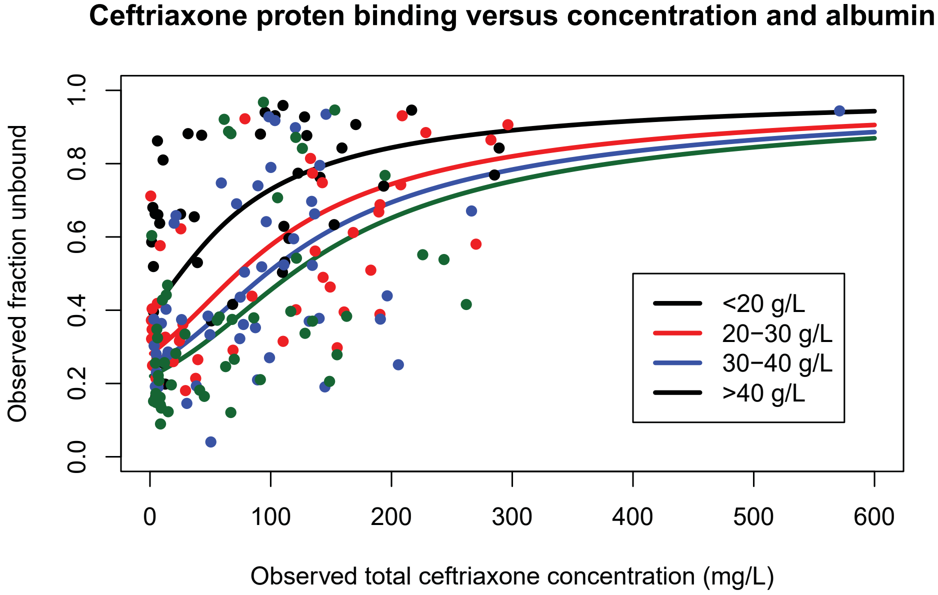

Figure S10: Comparison of observed ceftriaxone fraction unbound and observed total concentration with model-predicted changes with concentration and albumin

1.5 Ceftriaxone model details

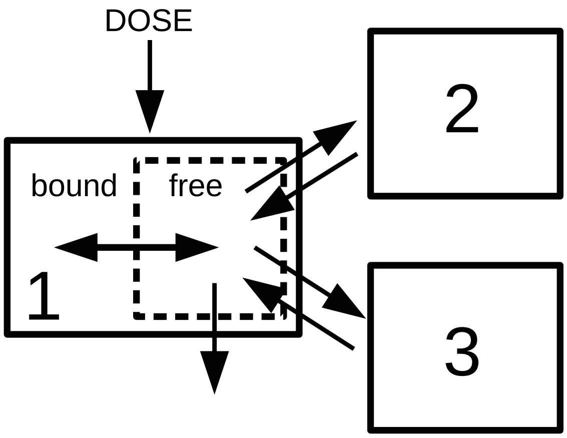

Figure S11: Schematic of the ceftriaxone model

The ceftriaxone dose is administered into compartment 1. Assuming instantaneous binding, unbound contentration (C_U) is then predicted from total concentration (C_T) as described in the Methods. Fraction unbound (F_U) is then calculated from C_U/C_T. The amount leaving compartment 1, either due to elmination or distribution, is multipled by F_U in order that only free drug is available for elimination or distribution. Differential equations relating to amounts of drug in each compartment are:

$$\frac{dA_1}{dt} = -A_1 F_U (k + k_{12} + k_{13}) + k_{21} A_2 + k_{31} A_3$$
$$\frac{dA_2}{dt} = k_{12} A_1 F_U - k_{21} A_2$$
$$\frac{dA_3}{dt} = k_{13} A_1 F_U - k_{31} A_3$$

where $k = CL/V_1$, $k_{12} = Q_2/V_1$, $k_{13} = Q_3/V_1$, $k_{21} = Q_2/V_2$, and $k_{31} = Q_3/V_3$. V_1 is therefore the total ceftriaxone central volume of distribution, whereas V_2 and V_3 are the periphearal free volumes, CL is the free ceftriaxone clearance, and Q_2 and Q_3 are the free intercompartmental clearances.

2 NONMEM model files

2.1 Ceftriaxone NONMEM model code

```
$PROBLEM    Ceftriaxone FLACSAM modelling
$INPUT      ID TIME TAFDM AMT DRUG DV EVID WT PMA AGED PRET SEX ALB
            SECR TPROT SITE OEDM CMTD EXCL L2
$DATA      FLACSAM_pk_cef_170102.csv IGNORE=@
$SUBROUTINE ADVAN6 TOL=5
$MODEL      COMP=(TOTAL) COMP=(PREFREE1) COMP=(PERFREE2)
$PK
;--- Typical parameters
 TVCL  = THETA(1)
 TVV1  = THETA(2)
 TVQ2  = THETA(3)
 TVV2  = THETA(4)
 TVQ3  = THETA(5)
 TVV3  = THETA(6)
 TVBM  = THETA(7)
 TVKD  = THETA(8)
;--- MU Modelling
 MU_1  = DLOG(TVCL)
 MU_2  = DLOG(TVV1)
 MU_3  = DLOG(TVBM)
;--- Mean expected creatinine for age
AGEY   = AGED/365.25
MSCR   = -2.37330-12.91367*DLOG(AGEY)+23.93581*AGEY**0.5  ; Mean SeCr, age adjusted  (F. Ceriotti et al,
;--- Oedema weight correction
IF(OEDM==0) WTC = WT
IF(OEDM>0)  WTC = WT * (1 + THETA(11))
;--- Individual parameters
 CL  = DEXP(MU_1 + ETA(1)) * (WT/70)**0.75 * PMA**3.4/(PMA**3.4 + 47.7**3.4) * (SECR/MSCR)**THETA(10)
 V1  = DEXP(MU_2 + ETA(2)) * (WTC/70)
 Q2  = TVQ2 * (WT/70)**0.75
 V2  = TVV2 * (WTC/70)
 Q3  = TVQ3 * (WT/70)**0.75
 V3  = TVV3 * (WTC/70)
 BM  = DEXP(MU_3 + ETA(3)) * (ALB/33.75)**THETA(9)
 KD  = TVKD
;--- Rate constants
 K    = CL/V1
 K12 = Q2/V1
 K21 = Q2/V2
 K13 = Q3/V1
 K31 = Q3/V3
;--- Time after dose
IF(EVID==1) TM  = TIME
IF(EVID==1) TAD = 0
IF(EVID/=1) TAD = TIME-TM

$DES
 CTOT   = A(1)/V1
```

```
 CFREE = 0.5*((CTOT-BM-KD) + SQRT((CTOT-BM-KD)**2 + 4 * KD * CTOT))
 FU    = CFREE/CTOT
 DADT(1) = - K*A(1)*FU - K12*A(1)*FU + K21*A(2)  - K13*A(1)*FU + K31*A(3)
 DADT(2) = K12*A(1)*FU - K21*A(2)
 DADT(3) = K13*A(1)*FU - K31*A(3)

$ERROR
 IPRED = 0
 CTOTE = A(1)/V1
 CCFRE = 0.5*((CTOTE-BM-KD) + SQRT((CTOTE-BM-KD)**2 + 4 * KD * CTOTE))
 CBOUN = CTOTE - CCFRE
 IF(DRUG==0.2) IPRED = CCFRE
 IF(DRUG==0.1) IPRED = CTOTE
 IF(DRUG==0.2) Y     = IPRED*(1 + EPS(1)) + EPS(2)
 IF(DRUG==0.1) Y     = IPRED*(1 + EPS(3)) + EPS(4)
;

$THETA  (0,4.61627) ; 1. TVCL
$THETA  (0,14.4644) ; 2. TVV1
$THETA  (0,0.933031) ; 3. TVQ2
$THETA  (0,6.05411) ; 4. TVV2
$THETA  (0,29.9815) ; 5. TVQ3
$THETA  (0,19.2606) ; 6. TVV3
$THETA  (0,69.7743) ; 7. TVBM
$THETA  (0,22.8853) ; 8. TVKD
$THETA  0.555443    ; 9. ALB power cov
$THETA  -0.261234   ; 10. SECR power cov
$THETA  (-1,-0.255959) ; 11. WT correction for any oedema
;
$OMEGA  BLOCK(2)
 0.174903
 0.0807096 0.18196
$OMEGA  0.172225
;
$SIGMA  0.0710923
$SIGMA  0  FIX
$SIGMA  0.0362471
$SIGMA  0.05
;
$ESTIMATION METHOD=1 INTER MAXEVAL=9999 PRINT=1
;
$COVARIANCE
$TABLE      ID PRED IPRED AMT TIME CWRES DV NPDE TAD CFREE CBOUN
            NOPRINT ONEHEADER FILE=sdtab33
$TABLE      WT PMA AGED PRET SEX ALB SECR TPROT DRUG OEDM WTC CTOT
            NOPRINT ONEHEADER FILE=cotab33
$TABLE      CL V1 Q2 V2 Q3 V3 BM KD ETAs(1:3) NOPRINT ONEHEADER
            FILE=patab33
```

2.2 Metronidazole NONMEM model code

```
$PROBLEM    Metronidazole FLACSAM modelling
$INPUT      ID TAFDC TIME AMT DRUG DV EVID WT PMA AGED PRET SEX ALB
            SECR TPROT SITE OEDM HCT AST CMT RATR EXCL L2
$DATA      FLACSAM_pk_met_170104.csv IGNORE=@
$SUBROUTINE ADVAN7 TRANS1
$MODEL      COMP=(DEPOTPAR) COMP=(DEPOTMET) COMP=(PARENT) COMP=(METAB)
$PK
;--- Typical parameters
 TVKA  = THETA(1)
 TVCLI = THETA(2)
 TVCL  = THETA(3)
 TVV1  = THETA(4)
 TVCLM = THETA(5)
 TVV2  = THETA(6)
 TVAL1 = THETA(7)
;--- MU Modelling
 MU_1 = DLOG(TVKA)
 MU_2 = DLOG(TVCLI)
 MU_3 = DLOG(TVCL)
 MU_4 = DLOG(TVV1)
 MU_5 = DLOG(TVCLM)
 MU_6 = DLOG(TVV2)
;--- Age years for CYP2A6 maturation function
AGEY = AGED/365.25
;--- Oedema weight correction
IF(OEDM<=1) WTC = WT
IF(OEDM>1)  WTC = WT * (1 + THETA(8))
;--- Individual parameters
 KA  = DEXP(MU_1 + ETA(1)) * (WT/70)**(-0.25)
 CLI = DEXP(MU_2 + ETA(2)) * (WT/70)**0.75 * ((1.1 - 0.15) * AGEY**0.8/(0.04**0.8 + AGEY**0.8) + 0.15)
 CL  = DEXP(MU_3 + ETA(3)) * (WT/70)**0.75
 V1  = DEXP(MU_4 + ETA(4)) * (WTC/70)
 CLM = DEXP(MU_5 + ETA(5)) * (WT/70)**0.75  * PMA**3.4/(PMA**3.4 + 47.7**3.4)
 V2  = DEXP(MU_6 + ETA(6)) * (WTC/70)
 ALAG1 = TVAL1
 ALAG2 = ALAG1
;--- Hepatic metabolism
QH   = 76 * (WT/70)**0.75     ; Hepatic blood flow
QHP  = QH * (1 - HCT * 0.91)  ; Hepatic plasma flow
ER   = CLI/(QHP + CLI)        ; Extraction ratio
CLTM = QHP*ER
F1   = 1-ER
F2   = ER
;--- Rate constants
 K13 = KA
 K24 = KA
 K30 = CL/V1
 K40 = CLM/V2
 K34 = CLTM/V1
;--- Time after dose
IF(EVID==1) TM  = TIME
```

```
IF(EVID==1) TAD = 0
IF(EVID/=1) TAD = TIME-TM

$ERROR
 IPRED = 0
 IF(DRUG==1.1) IPRED = A(3)/V1
 IF(DRUG==1.2) IPRED = A(4)/V2
 IF(DRUG==1.1) Y     = IPRED*(1 + EPS(1)) + EPS(2)
 IF(DRUG==1.2) Y     = IPRED*(1 + EPS(3)) + EPS(4)
;
$THETA  (0,0.393084) ; 1. TVKA
$THETA  (0,0.743542) ; 2. TVCLI
$THETA  (0,0.505611) ; 3. TVCL
$THETA  (0,56.3524) ; 4. TVV1
$THETA  (0,1.45238) ; 5. TVCLM
$THETA  (0,16.8914) ; 6. TVV2
$THETA  (0.1,0.171638,0.75) ; 7. AL1
$THETA  (-1,-0.16871) ; 8. Oedema cov
;
$OMEGA  0.626046
$OMEGA  0.376697
$OMEGA  0  FIX
$OMEGA  0.141027
$OMEGA  0  FIX
$OMEGA  0  FIX
;
$SIGMA  0.104557
$SIGMA  0  FIX
$SIGMA  0.0829706
$SIGMA  0.0149359
;
$ESTIMATION METHOD=1 INTER MAXEVAL=9999 PRINT=1
;
$COVARIANCE
$TABLE      ID PRED IPRED AMT TIME CWRES DV NPDE TAD CMT NOPRINT
            ONEHEADER FILE=sdtab130
$TABLE      WT PMA AGED PRET SEX ALB SECR TPROT DRUG OEDM AST NOPRINT
            ONEHEADER FILE=cotab130
$TABLE      KA CLI CL V1 CLM V2 F1 ER CLTM ETAS(1:6) K13 K24 K30 K40
            K34 NOPRINT ONEHEADER FILE=patab130
```

3 Metronidazole model identifiability and parameter interpretation

The well stirred model has been used to predict an apparent extraction ratio, by estimating an intrinsic clearance and fixing hepatic plasma flow. Our median extraction ratio was `0.015`, and the median fraction metabolised (FM), calculated by taking the ratio of clearance to metabolite to total parent clearance (FM = CLTM / (CLTM + CL)), was estimated to be `0.636`.

It should be noted however that our modelling did not aim to draw firm conclusions on mechanistic or physiologically-based pharmacokinetic factors such as this, and we have consciously ignored issues such as possible enterohepatic recirculation and whether a fraction unbound in blood needs to be added to capture a

true intrinsic clearance. Intravenous dosing (to separate out fraction absorbed plus fraction metabolised in the gut wall from fraction metabolised in the liver) and urinary data of parent and metabolite concentrations would ideally have been required to do this, neither of which were feasible to collect in the clinical setting of treating small intestinal overgrowth in children with severe acute malnutrition.

Our reason however for using the well-stirred model was the desire to allow metabolite formation during both first-pass and circulating parent, which as discussed in the manuscript improved model fit, making our model useful for its purpose of predicting $ft > MIC$ and $AUC_{(0-24)}/MIC$. To allow first pass and circulating metabolite formation necessitated fixing absorption rate constant to be the same for parent and metabolite, and also fixing one of clearance to metabolite, metabolite volume or metabolite clearance for example. The well-stirred model provides a convenient mechanism for doing this in that if one assumes all formation during first-pass is due to hepatic metabolism and fix the clearance to metabolite as hepatic flow multiplied by extraction ratio, the model becomes structurally identifiable. Since this is not a commonly used approach in population parent-metabolite pharmacokinetic modelling, in addition to the model code provided above, below details are given on structural and practical identifiability.

3.1 Structural model identifiability

This was checked using the COMBOS web application available at http://biocyb1.cs.ucla.edu/combos/, and cited in:

Meshkat N, Kuo CE, DiStefano J 3rd. On finding and using identifiable parameter combinations in nonlinear dynamic systems biology models and COMBOS: a novel web implementation. PLoS One. 2014 Oct 28;9(10):e110261.

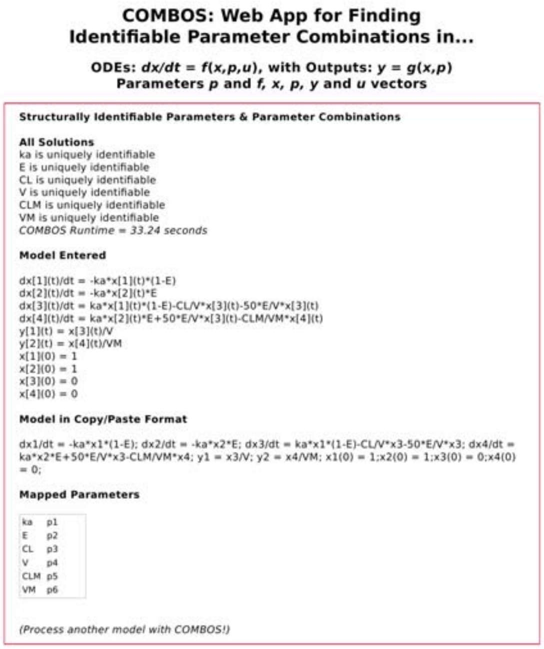

Figure S12: COMBOS identifiability result

The result was that the absorption rate constant (`ka`), extraction ratio (`E`, derived from estimating intrinsic clearance combined with fixed liver plasma flow in our model, and multiplied by fixed hepatic plasma flow to derive clearance to metabolite `CLTM`), parent clearance and volume (`CL` and `V`), and metabolite clearance and volume (`CLM` and `VM`), are structurally identifiable. In the COMBOs software code above we use 50 as an apporximate hepatic plasma flow for illustration of where `CLTM` comes in.

3.2 Practical identifiability

The following steps were taken to assess practical identifiability: Firstly to test sensitivity to initial estimates, the model was re-run 10 times with random initial perturbations of up to 80% (using the PsN command `execute run130.mod -tweak_inits -degree=0.8 -min_retries=10`); Secondly log-likelihood profiles were generated for each parameter (using the PsN command: `llp run130.mod -thetas=1,2,3,4,5,6 -dir=run130llp`). Practical identifiability was then defined as final parameter estimates and objective function value being similar across initial estimate perturbations, and log-likelihood profiles showing clear minimum (as opposed to flat trajectories) for all parameters. The results were as follows:

Parameter estimate perturbation result (all models having minimised successfully with successful covariance step):

Model	OFV	THETA1	THETA2	THETA3	THETA4	THETA5	THETA6	THETA7	THETA8
original	2285.726	0.393084	0.743542	0.505611	56.3524	1.45238	16.8914	0.171638	-0.168710
rep 1	2285.726	0.393094	0.743521	0.505635	56.3523	1.45234	16.8907	0.171645	-0.168696
rep 2	2285.726	0.393100	0.743518	0.505612	56.3517	1.45235	16.8909	0.171641	-0.168702
rep 3	2285.726	0.393085	0.743538	0.505615	56.3521	1.45238	16.8913	0.171641	-0.168711
rep 4	2285.726	0.393072	0.743533	0.505631	56.3529	1.45236	16.8908	0.171620	-0.168706
rep 5	2285.726	0.393091	0.743514	0.505642	56.3525	1.45233	16.8907	0.171643	-0.168710
rep 6	2285.726	0.393095	0.743507	0.505647	56.3522	1.45233	16.8905	0.171644	-0.168709
rep 7	2285.726	0.393079	0.743455	0.505664	56.3508	1.45230	16.8890	0.171630	-0.168695
rep 8	2285.726	0.393095	0.743541	0.505600	56.3525	1.45239	16.8914	0.171641	-0.168696
rep 9	2285.726	0.393104	0.743574	0.505571	56.3527	1.45245	16.8924	0.171638	-0.168718
rep 10	2285.726	0.393066	0.743467	0.505664	56.3526	1.45229	16.8896	0.171632	-0.168693

Log-likelihood profile of structural model parameters:

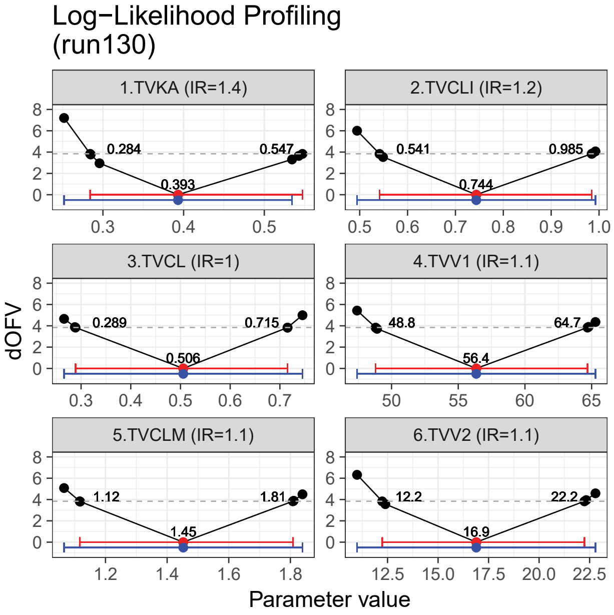

Figure S13: Log-likelihood profile from the structural parameters of the metronidazole model. X-axis gives parameter value, change in objective function value is on the y-axis. Confidence intervals computed from the standard error under Normal assumption (blue line) and log-likelihood profile (red line) are shown. The IR value gives a measure of log-likelihood profile symetry (equal to 1 it means symmetric)

In summary the preceding identifiability analysis suggests the model is robust to initial estimates and is structurally and practically identifiable.
